# Individual and community level factors associated with medical treatment-seeking behavior for childhood diarrhea among the Gambian mothers: evidence from the Gambian demographic and health survey data, 2019/2020

**DOI:** 10.1186/s12889-023-15493-2

**Published:** 2023-03-28

**Authors:** Bewuketu Terefe, Bezawit Mulat, Kegnie Shitu, Nega Tezera Assimamaw

**Affiliations:** 1 Department of Community Health Nursing, School of Nursing, College of Medicine and Health Sciences, University of Gondar, Gondar, Ethiopia; 2 Department of Human Physiology, School of Medicine, College of Medicine and Health Sciences, University of Gondar, Gondar, Ethiopia; 3 Department of Health Education and Behavioral Sciences, Institute of Public Health, of Medicine, College of Medicine and Health Sciences, University of Gondar, Gondar, Ethiopia; 4 Department of Pediatrics and Child Health, School of Nursing, College of Medicine and Health Sciences, University of Gondar, Gondar, Ethiopia

**Keywords:** Diarrhea, Childhood diarrhea, Individual and community level factors, Medical treatment seeking behaviors, Gambia

## Abstract

**Introduction:**

In less developed countries, including the Gambia, childhood diarrhea is one of the leading causes of serious illness and death among children. Studies on wider determinants of behaviors in medical treatment seeking for diarrheal illnesses in poor resource settings are limited. However, the challenges are continuing and, there is a gap in research work about it in the Gambia. Therefore, the rationale of this study was to assess the individual and community level factors of medical treatment-seeking behaviors for childhood diarrhea among mothers in the Gambia.

**Methods:**

Data from the 2019–20 Gambia demographic and health survey were used in this study, which was based on secondary data analysis. A total of 1,403 weighted samples of under-five children’s mothers were included in the study for diarrhea medical treatment-seeking behaviors. Because of the hierarchical nature of the data, a multi-level logistic regression model was applied to identify individual and community-level factors that may influence mothers’ medical treatment-seeking behaviors of diarrhea. Data were analyzed using multilevel logistic regression analysis. In the multivariable multilevel logistic regression analysis, variables were judged significantly linked with medical treatment-seeking behavior of diarrhea if their p-value was less than 0. 05.

**Results:**

Medical treatment-seeking behaviors for diarrhea were discovered in 62.24% (95% CI: 59.67,64.74) of mothers of under five children. Being a female child has shown odds of (AOR = 0.79, (CI 95%: (0.62,0.98)) times less treatment-seeking behavior than the counterparts. Moreover, compared to mothers whose children were of average size, those whose children were smaller, and larger than average at birth were more likely to seek out pediatric medical treatment (AOR = 1.53, 95% CI (1.08–2.16), and (AOR = 1.31, 95% CI (1.01,1.169)) respectively. On the other side, mothers who have exposure to listening to the radio, and heard about oral rehydration have shown an odds of (AOR = 1.34, CI 95%, (1.05,1.72)), (AOR = 2.21, CI 95%, (1.14,4.30)), being from the middle, and rich household wealth have also shown (AOR = 2.15, CI 95%, (1.32,3.51)), and (AOR = 1.92, (CI 95%, (1.11,3.32)), a child with cough, and fever (AOR = 1.44, CI 95%, (1.09,1.89)), and (AOR = 1.73, CI 95%, (1.33,2.25)) were individual-level factors that have shown association statistically with the outcome variable. Similarly, regarding community level factors mothers who had a postnatal checkup, and those from the Kerewan region have revealed more odds of (AOR = 1.48, CI 95%, (1.08,2.02)), and (AOR = 2.99, CI 95%, (1.32,6.78)) times significantly with treatment seeking behavior of mothers respectively.

**Conclusion:**

Diarrhea medical treatment-seeking behavior was found low. Hence, it remains among the top public health challenges in the Gambia. Strengthening mothers’ healthcare-seeking behavior and skills on home remedies, and childhood illnesses, advocating mass media exposure, assisting financially disadvantaged mothers, and postnatal checkups after delivery will enhance medical treatment-seeking behavior. Furthermore, coordinating with regional states, and designing timely policies and interventions are highly advisable in the country.

## Introduction

Diarrhea is defined as three or more loose or watery stools in 24 h, or an increase in a person’s daily stools’ fluidity, frequency, or volume relative to what is regarded as [1, 2]. Inadequate personal hygiene, tainted food, and shared water can all contribute to diarrhea, just like other infectious [2]. It is a leading cause of sickness and mortality in children under the age of five worldwide, killing an estimated 533,768 children under the age of five in 2017 [3, 4]. According to a report from the World Health Organization (WHO), diarrhea causes 1.7 million child infections and is the second most common cause of death for children under the age of five worldwide. However, only sub-Saharan Africa and south Asia account for about 80% of all child morbidity and mortality. Many other factors are important in these areas, but malnutrition, which exposes youngsters to diarrhea, accounts for a large portion of the [2, 5]. Centers for disease control and prevention also reported that diarrhea claims the lives of 2,195 children every day, which equates to approximately 32 school buses full of kids every day. This is even worse than the combined prevalence of AIDS, malaria, and [6]. Infection, starvation, and illiteracy exacerbate the situation in impoverished countries among under-five [2, 7]. Dehydration, which can be treated with an oral rehydration solution (ORS), is the leading cause of diarrheal death. ORS, on the other hand, is unable to reduce the volume, frequency, or duration of diarrhea on its [8].

Several studies have been undertaken to examine the determinants of mothers’ health-seeking behaviors for diarrhea disease in their children, and various factors have been identified. Structure factors such as distance to health [9], cultural beliefs, [10], and mother’s [11] are among them, as are socio-demographic determinants such as media exposure, and [12], mother’s education [13], mother’s marital [14, 15], child’s [14], and child’s [16, 17].

Clinical studies have demonstrated that combining zinc and ORS can help lower morbidity and mortality [7]. According to the World Health Organization (WHO) and United Nations Children’s Fund (UNICEF), oral rehydration salts, zinc supplements, and extended feeding are recommended to treat childhood diarrhea [18]. In developing nations, less than 40% of children with diarrhea receive a prescription for treatment, and little has been done to change this [19]. Excellent diarrhea treatment in Africa is rare, with rates ranging from 17% in Cote d’Ivoire to 38% in Niger. This treatment includes oral rehydration therapy, zinc, and more frequent [20]. The Clinton Health Access Initiative (CHAI) collaborated with governments, medical professionals, and communities in India and a few African countries from 2012 to 2016 to improve the treatment of diarrhea. The effort was successful in increasing the combined use of zinc and ORS in several of its target [21]. The Integrated Management of Neonatal and Childhood Illness (IMNCI) suggests using these two drugs to treat acute diarrhea.

Reports show that, even though tremendous activities have been done to reduce the severity of diarrhea, it is still among the upmost leading causes of death for under-five children in the Gambia next to malaria and respiratory [22, 23]. Oral rehydration for diarrheal disorders could have saved the lives of the majority of these [10, 17]. However, in developing nations, many children’s lives are still lost as a result of ineffective treatment or failure to seek health care from health facilities, as well as delays in seeking health care by [10, 24]. In the Gambia, mothers’ health-seeking behavior is likewise unappealing, with only a few sick under-five-year-old children being treated at health [25]. Empirical research suggests that if proper health care is obtained, disease burden and fatalities from common childhood illnesses like diarrhea can be significantly [26]. In underdeveloped nations, where a considerable proportion of children continue to die from childhood febrile diseases, mothers’ capacity to recognize and seek proper health treatment is critical in decreasing child [10, 27]. The cornerstone of Agenda 2063 is Africa’s youth, especially its children, and it states that two thirds of under-five mortality are caused by preventable causes, primarily pneumonia, malaria, diarrheal diseases, measles, and HIV/AIDS, the majority of which are made worse by [28]. Therefore, this study will have its own positive contribution the African agenda 2063.

To the best of our literature knowledge, nothing data is recorded in the Gambia on under-five children’s treatment-seeking behaviors regarding the diarrheal disease. Therefore, by identifying the pattern of treatment-seeking behavior of mothers and individual and community level determinants, this study will provide preliminary nationwide information to the government, stakeholders, health institutions, and other concerned bodies of stakeholders who are interested in working in maternal and children’s health in the Gambia.

## Method and materials

### Study setting and period

The Gambia is a long, narrow country that juts out 487 km into the interior of the continent of Africa. Its average width is 24 km. The country is wider than 48 km, or twice the normal distance, where the river Gambia meets the Atlantic Ocean. Senegal borders the nation on three sides, and the Atlantic Ocean borders it on the west. The Gambia has a land size of 10,689.28 km2, making it one of the tiniest countries in West Africa. An estimated 1.85 million people called the Gambia home in 2013, and more than half of them resided in urban and semi-urban areas. The current evidence shows that Gambia has 38.18/1000, and 57.8/1000 live births infant mortality and deaths of children under five years old respectively. In the Gambia, there is going to be 92.36 thousand births in 2021. It rounds out to 253 daily, which ranks 112th. 37.09 births per 1,000 people is the crude birth rate. In Gambia, there will be 18.37 thousand fatalities in 2021. That amounts to 50 each day, which ranks 144th. Among 1000 people, there are 7.38 deaths on average. The ratio of female to male is practically [29]. The majority of the population are Muslims (95%), and there is a small proportion of Christians (4%) and followers of other indigenous religions. The survey was conducted between November 21, 2019, and March 30, 2020 [30].

### Data source, population, and sampling procedure

Data from the most recent Gambian Demographic and Health Survey (GDHS) for the years 2019-20 were used in this study. The survey was conducted between November 21, 2019, and March 30, 2020. For this survey, a complete list of 4,098 Enumeration Areas (EAs) was provided by the Gambian Population and Housing Census. The stratified two-stage cluster sampling method was utilized. In the first round, 281 EAs were chosen. In the second step, a total of 25 households per cluster/EA were picked. The data is available to the general public. We were able to obtain the dataset utilized in this inquiry after registering and receiving an authentication letter from the Demographic and Health Survey (DHS) program at the DHS Program - Gambia: Standard DHS, 2010-20 [30]. Next this, a weighted total number of 1,403 mothers and under-five children paired were included in the study to determine again the treatment-seeking behavior of diarrhea among the study population in detail.

#### Inclusion and exclusion criteria

Those children under five who had diarrhea within the two weeks prior to the survey were included, while those who had not experienced diarrhea were left out of the study.

### Variables of the study

#### Outcome variable

Medical treatment-seeking behavior for childhood diarrhea among mother was the study’s outcome variable. The variable was dichotomized, with 0 “do not have medical treatment seeking behavior for diarrhea” and 1 “have medical treatment seeking behavior for diarrhea”. To calculate the sample size for this investigation, we first calculated the prevalence of diarrhea among all under-five survey participants, a weighted sample of 7,298 children, of which 1,403 weighted children had diarrhea. Because the goal of this study was to evaluate mothers’ medical treatment-seeking behavior and its contributing factors, we omitted any children who had not had diarrhea two weeks prior to the survey. Finally, we included 1,403 paired mothers and their under-five children in our sample size.

#### Independent variables

In this study, many factors from individual and community levels were included. Sex of the child, the current age of a child, number of health visits, visits to a health facility in the last 12 months, visited by a field health worker in the last 12 months, order of birth, twins of a child, place of delivery, birth weight, duration of breastfeeding, maternal age, religion, maternal, and husband educational levels, ethnicity, mass media exposure (watching to television, listening to the radio and reading to magazine/newspapers), number of household members, household wealth, marital status, maternal, and husband occupation status, the total number of children ever born, number of under-five children, mother heard of oral rehydration, a child had cough and fever, and covered by health insurance were considered as individual-level factors. On the other hand, place of residence, region, community-level women’s education, community level women’s wealth status, community level media exposure, community level distance to health facility, community level postnatal check, and community heard family planning from health care providers were among variables of community-level factors respectively.

## Operational definitions

All the community levels factors were calculated concerning their aggregated variables because they were neither observable nor documented during the survey. Each of them computed respective of their individual variable value, however, the procedure is the same like other literatures. A community level factor in this research was defined as a collection of households that shared a primary sample unit or cluster in the dataset. Aggregating elements at the individual level to create variables at the community level. The community variables included region, place of residence, community mass media exposure status (proportion of mothers in the community who were exposed for radio or television exposure at least once a week), community postnatal checkup (proportion of mothers in the community who were checked up), community education (proportion of mothers in the community with primary/post primary education), community wealth (proportion of mothers in the community who were rich), and community distance to a health facility (proportion of mothers in the community who perceived the distance to a health facility as a problem). For ease of results interpretation, the continuous community-level variables were further divided into low and high categories using the mean/median value based on their [31–33].

### Community level women’s education

This is the aggregate value of the educational levels of women based on the average proportions of educational levels in the community. It was defined as low if the ratio of women with secondary education & above in the community was below the median and high if the value is higher than the median. The median value was 0.08.

### Community level women’s wealth status

This variable is also derived from an individual household’s wealth index with the same procedure. It was defined as high if the proportion of women from the two lowest wealth quintiles in a given community was 64.71–100% and low if the proportion was 0–64.70%. The median value was 64.70.

### Community-level media exposure

This variable was derived from individual responses to radio or television exposure. It was defined as low if the proportion of women exposed to media in the community was 0–74% and high if the proportion was 75–100%. The median value was 74%.

### Community level distance to health facility

The variable aggregated from individual perceived distance to a health facility is a big problem. It was categorized as low if the proportion of women who perceived health facility distance as a big problem in the community was 0–16.67% and categorized as high if the proportion was between 16.68% and 100%. The median value was 16.67%.

### Community postnatal checkup

This variable was aggregated from an individual’s postnatal checkup after delivery. It was classified as low if the value is below the mean value of 0-50.82% and high if it is between 50.83 and 100%. The mean value was 50.83%.

### The community heard about family planning from health care providers

Similarly, this variable was aggregated from individual variables related to hearing about family planning from health care providers at any time before the survey. It was classified as low if the value is below the mean value of 0-50.82% and high if it is between 50.83 and 100%. The mean value was 50.83%.

### Data processing, procedure, and analysis

Data were extracted from Kids Records (KR) files, coded, and processed using STATA version 14 statistical software, and excel. Weighted samples were utilized for the analysis to consider the varied chances of selection and non-response in the initial survey. Due to the use of multi-stage stratified cluster sampling techniques, the data from the Gambian demography and health survey (GDHS) have a hierarchical structure. Single-level logistic regression is not recommended in this scenario since conventional multilevel logistic regression algorithms treat the units of analysis as independent observations. Due to an inability to recognize hierarchical patterns, regression standard errors will be overstated, which will result in an overestimation of statistical significance. An advanced statistical model that considers the hierarchy of the data is necessary to draw significant deductions and conclusions. To evaluate the fixed and random effects of the factors linked to treatment seeking behavior of mothers, a multivariable multi-level logistic regression model was [34].

A multilevel model analysis method which consists of both individual and community level factors was employed to determine the treatment-seeking behavior of mothers among exposed under five children. A total of four models were created. The first model was fitted without any explanatory variables and is known as an empty or null model. The purpose of this approach was to break down the variance between communities. Understanding community variances necessitates the use of the null model. We utilized it as a baseline to determine how much community factors could account for the observed differences in mothers’ behavior on treatment seeking. Furthermore, as a litmus paper for whether multi-level or standard logistic regression should be utilized, this model was used to justify the usage of a multi-level statistical framework. The Log-Likelihood Ratio test (LLR), Median Odds Ratio (MOR), Intra-class Correlation Coefficient (ICC), and Proportional Change of Variance (PCV) were used to evaluate it, and AIC were applied. Only individual-level characteristics were included in the second model. Only community-level traits were present in the third. In comparison, the final (fourth) model included both individual and community-level factors. Moreover, the model comparison was made using model deviance, a model with the lowest deviance selected for reporting and interpretation results. The cutoff criteria for choosing variables for the multi-variable analysis entry was a p-value of less than 0.2. The Gambia employed multivariable multilevel logistic regression to isolate independent determinants of diarrhea treatment-seeking behavior while adjusting confounders. The statistical significance was assessed using the 95% confidence interval (CI) and a p-value of 0.05.

## Results

### Individual level sociodemographic characteristics study population on treatment seeking behavior of diarrhea

In this survey, 1,403 weighted samples of paired mothers and under-five children who experienced diarrhea were considered for analysis. 765(54.51%) children were male. In this survey, for Current age of the child in a year, order of birth, birth weight, and the number of under-five children were described with a proportion of 822(58.62) were from 0 to 1 years, from 2-4th birth order 688(49.05), average birth weight 594(42.36), and 806(57.43) of them were found from family’s members of greater than ten respectively. In this study, concerning age, wealth status, marital status, working status, educational background, and religious affiliation, 731(52.08), 328(23.34), 1,276(90.98), 916(65.30), 618(44.09), and 1,371(97.75) of them were poorest, married, currently have work, had no enrolled formal education and practicing Islamic religion respectively. In the same pattern of expression, 800(57.03), and 1,359(96.85) husbands/partners were educated and had work currently respectively. To place of delivery (1,212(86.38), number of children born 818(58.34), media exposure of, television (733(52.26), radio 567(40.42) and ethnicity 394(28.06) were delivered in the health facility, from 1 to 3 children per household, watching television, listening radio and from Mandinka/Jahanka ethnic group respectively (Table 1).

**Table 1:** Individual-level variables descriptive result of treatment-seeking behavior of diarrhea among mothers/caregivers of under-five children in Gambia, GDHS 2019/20 (n = 1,403)

Variables	Diarrhea treatment-seeking behavior		Total,n (%)
	No, n (%)	Yes, n (%)	
Sex of the child rea			
Male	277(36.27)	487(63.73)	765(54.51)
Female	252(39.55)	386(60.45)	638(45.49)
The current age of the child in the year			
0–1	298(36.29)	524(63.71)	822(58.62)
2–3	189(40.46)	278(59.54)	467(33.32)
4–5	42(37.33)	71(62.67)	113(8.07)
Number of health visits			
1–2	483(37.67)	798(62.33)	1,281(91.30)
3–5	47(38.71)	75(61.29)	122(8.70)
They visited a health facility in the last 12 months			
No	53(38.91)	84(61.09)	137(9.77)
Yes	476(37.64)	789(62.36)	1,266(90.23)
Visited by a field health worker			
No	441(38.84)	695(61.16)	1,136(80.98)
Yes	89(33.18)	178(66.82)	267(19.02)
Order of birth			
First	137(40.93)	198(59.07)	335(23.89)
2–4	245(35.60)	443(64.40)	688(49.05)
Five and above	148(38.89)	232(61.11)	380(27.06)
Does a child is twin			
No(single)	514(37.96)	840(62.04)	1,355(96.58)
Yes(multiple)	15(32.09)	33(67.91)	48(3.42)
Place of delivery			
Home	70(36.55)	121(63.45)	191(13.62)
Health facility	460(37.95)	752(62.05)	1,212(86.38)
Birth weight			
Larger than average	205(35.05)	381(64.95)	586(41.79)
Average	245(41.21)	349(58.79)	594(42.36)
Smaller than average	79(35.68)	143(64.32)	222(15.86)
Duration of breastfeeding			
Ever breastfed/not currently breastfed	273(39.90)	412(60.10)	685(48.86)
Never breastfed	8(41.40)	10(58.60)	18(1.30)
Still breastfed	249(35.57)	450(64.43)	699(49.84)
Maternal age			
15–24	157(40.67)	229(59.33)	387(27.56)
25–34	263(36.01)	467(63.99)	731(52.08)
35–49	109(38.30)	176(61.70)	286(20.36)
Religion			
Islam	518(37.74)	854(62.26)	1,371(97.75)
Christian	12(38.60)	19(61.40)	32(2.25)
Mothers’ highest education level			
No formal education	240(38.75)	379(61.25)	618(44.09)
Primary education	106(36.92)	181(63.08)	287(20.47)
Secondary education	167(37.19)	282(62.18)	449(31.98)
Higher education	17(35.43)	31(64.57)	49(3.46)
Husband/partner’s education level			
No educated	205(34.05)	397(65.95)	603(42.97)
Educated	324(40.56)	476(59.44)	800(57.03)
Ethnicity			
Mandinka/Jahanka	167(42.30)	227(57.70)	394(28.06)
Wollof	67(32.89)	136(67.11)	203(14.44)
Jola/Kasroninka	58(48.23)	63(51.77)	121(8.61)
Fula/Tukulur/Lorobo	103(34.27)	198(65.73)	301(21.48)
Serere	10(25.47)	28(74.53)	38(2.71)
Sarahule	33(31.68)	70(68.32)	103(7.31)
Creole/Aku/Marabout	0(0)	2(100.0)	2(0.14)
Manjago	4(21.41)	16(78.59)	20(1.40)
Bambara	6(33.50)	13966.50)	19(1.32)
Others	3(37.54)	5(62.46)	8(0.56)
Non-Gambian	80(40.61)	116(59.39)	196(13.98)
Mass media exposure			
Watching Television			
No	248(37.02)	422(62.98)	670(47.74)
Yes	282(38.44)	451(61.56)	733(52.26)
Listening to Radio			
No	330(39.44)	506(60.56)	836(59.58)
Yes	200(35.28)	367(64.72)	567(40.42)
Reading to newspaper/magazine			
No	520(37.77)	856(62.23)	1,376(98.06)
Yes	10(37.64)	7(62.36)	27(1.94)
Number of household members			
1–5	69(40.55)	101(59.45)	170(12.09)
6–10	154(36.06)	274(63.94)	428(30.48)
= >11	307(38.08)	499(61.92)	806(57.43)
Wealth index			
Poorest	122(37.18)	206(62.82)	328(23.34)
Poorer	114(38.73)	180(61.27)	293(20.89)
Middle	76(28.38)	191(71.62)	266(18.98)
Rich	116(40.06)	174(59.94)	290(20.70)
Richer	103(45.46)	123(54.54)	226(16.09)
Marital status			
Single/widowed/divorced	67(52.69)	60(47.31)	127(9.02)
Married	463(36.28)	813(63.72)	1,276(90.98)
Mothers’ occupation			
Current not working	193(39.72)	294(60.28)	487(34.70)
Currently working	336(36.72)	580(63.28)	916(65.30)
Husbands/partners’ occupation			
Currently not working	15(34.70)	29(65.30)	44(3.15)
Currently working	514(37.86)	844(62.14)	1,359(96.85)
Number of children ever born			
1–3	319(39.02)	499(60.98)	818(58.34)
Above 3	210(36.00)	375(64.00)	585(41.66)
Number of under-five children			
0–1	102(42.37)	139(57.63)	241(17.16)
2–3	236(36.59)	410(63.41)	646(46.06)
> 3	191(37.08)	325(62.92)	516(36.79)
Heard of oral rehydration			
No	25(56.54)	19(43.46)	44(3.14)
Yes	505(37.15)	854(62.85)	1,359(96.86)
Child had cough			
No	400(41.57)	562(68.43)	962(68.55)
Yes	130(29.47)	311(70.53)	441(31.45)
Child had fever			
No	395(41.79)	551(58.21)	946 (67.46)
Yes	134(29.41)	322(70.59)	457(32.54)
Covered by health insurance			
No	516(37.57)	857(62.43)	1,373(97.87)
Yes	14(46.65)	16(53.35)	30(2.13)

### Community level sociodemographic characteristics study population on treatment seeking behavior of diarrhea

A higher proportion of 943(67.24) participants were urban residents, of these 610(43.49) were belonging to the region Brikama. Similarly, 826(58.90), 880(62.69), 809(57.67), and 800(57.04) women were from communities with a high level of women’s education, low level of community wealth status, low level of media exposure, and low level of postnatal checkup in health facilities respectively. on the other hand, 845(60.26%) of them were not perceive that distance to a health facility is a big problem (Table 2).

**Table 2:** Community-level variables descriptive result of treatment-seeking behavior of diarrhea among mothers/caregivers of under-five children in Gambia, GDHS 2019/20(n = 1,403)

Variables	Diarrhea treatment-seeking behavior		Total, n (%)
	No, n (%)	Yes, n (%)	
Place of residence			
Urban	393(41.71)	550(58.29)	943(67.24)
Rural	137(29.67)	323(70.33)	460(32.76)
Region			
Banjul	8(47.49)	9(52.51)	17(1.22)
Kanifing	112(45.79)	132(54.21)	244(17.36)
Brikama	255(41.88)	355(58.12)	610(43.49)
Mansakonko	29(42.84)	39(57.16)	68(4.88)
Kerewan	22(17.42)	102(82.58)	124(8.86)
Kuntaur	36(29.61)	85(70.39)	121(8.66)
Janjanbureh	34(41.10)	48(58.90)	82(5.84)
Basse	34(25.02)	102(74.98)	136(9.70)
Community-level women education			
Low	184(31.89)	393(68.11)	577(41.10)
High	346(41.86)	480(58.14)	826(58.90)
Community-level women’s wealth status			
Low	349(39.69)	531(60.31)	880(62.69)
High	181(34.52)	342(65.48)	523(37.31)
Community-level media exposure			
Low	291(35.93)	518(64.07)	809(57.67)
High	239(40.25)	355(59.75)	594(42.33)
Community-level distance to the health facility			
Not a problem	330(39.10)	515(60.90)	845(60.26)
Problem	200(35.74)	358(64.26)	558(39.74)
Community postnatal check			
Low	352(43.94)	449(56.06)	800(57.04)
High	178(29.57)	425(70.43)	603(42.96)
The community heard family planning from HCPs			
Low	299(41.48)	422(58.52)	721(51.36)
High	231(33.84)	451(66.16)	682(48.64)

### Factors associated with treatment-seeking behavior of diarrhea

Mothers who have a female child have shown (AOR = 0.79, (CI 95%: (0.62,0.98)) times less likely to get health care treatment compared to participants with their counterpart parents. Moreover, compared to mothers whose children were of average size, those whose children were smaller, and larger than average at birth were more likely to seek out pediatric medical care (AOR = 1.53, 95% CI (1.08–2.16), and (AOR = 1.31, 95% CI (1.01,1.169)). On the other hand, mothers who have exposure to listening to the radio, and have heard about oral rehydration have shown (AOR = 1.34, CI 95%, (1.05,1.72)), and (AOR = 2.21, CI 95%, (1.14,4.30)) times more likely to take their children to a health facility at the time of diarrhea infection compared to their counterparts respectively. Furthermore, regarding the household wealth of middle and rich mothers have (AOR = 2.15, CI 95%, (1.32,3.51)), and (AOR = 1.92, (CI 95%, (1.11,3.32) times to take their children to the health facility to treat them than poorest household mothers respectively. Children who had cough and fever have shown a more likely treatment-seeking tendency to be taken to the health facility for their diarrhea than their counterparts by odds ratio (AOR = 1.44, CI 95%, (1.09,1.89)), and (AOR = 1.73, CI 95%, (1.33,2.25)) respectively. Similarly, regarding the community level factors mothers who have postnatal checkups and mothers from the region have shown a higher positive tendency to diarrhea treatment by the odds ratio of (AOR = 1.48, CI 95%, (1.08,2.02)), and (AOR = 2.99, CI 95%, (1.32,6.78)) than their counterparts respectively (Table 3).

**Table 3:** Bivariable and multivariable multilevel analysis of factors associated with seeking behavior of diarrhea among paired mothers and under-five children in the Gambia, GDHS 2019-20(n = 1,403)

Variables	Model null	Model I	Model II	Model III
	AOR (95% CI)	AOR (95% CI)	AOR (95% CI)	AOR (95% CI)
Sex of the child				
Male		1		1
Female		0.78(0.61,0.99)		0.79(0.62,0.98) *
The current age of the child in the year				
0–1		1		1
2–3		0.72(0.47,1.09)		0.77(0.51,1.17)
4–5		0.69(0.39,1.21)		0.69(0.39,1.21)
Birth weight				
Smaller than average		1.60(1.13,2.26)		1.53(1.08,2.16) *
Average		1.32(1.02,1.71)		1.31(1.01,1.69) *
Larger than average		1		1
Child had cough				
No		1		1
Yes		1.41(1.07,1.86)		1.44(1.09,1.89) *
Child had fever				
No		1		1
Yes		1.66(1.27,2.15)		1.73(1.33,2.25) *
Heard oral rehydration				
No		1		1
Yes		2.42(1.24,4.69)		2.21(1.14,4.30) *
Duration of breastfeeding				
Ever breastfed/not currently breastfed		0.46(0.11,1.96)		0.48(0.12,2.02)
Never breastfed		1		1
Still breastfed		0.40(0.09,1.72)		0.43(0.101,1.81)
Husband/partner education				
Not educated		1		1
Educated		0.81(0.62,1.05)		0.85(0.65,1.11)
Listening to radio				
No		1		1
Yes		1.37(1.07,1.75)		1.34(1.05,1.72) *
Wealth of household				
Poorest		1		1
Poorer		1.16(0.82,1.65)		1.32(0.91,1.91)
Middle		1.56(1.06,2.29)		2.15(1.32,3.51) *
Richer		1.27(0.84,1.91)		1.92(1.11,3.32) *
Richest		0.95(0.59,1.52)		1.63(0.88,2.99)
Under five children number				
0–1		1		1
2–3		1.22(0.87,1.72)		1.16(0.82,1.63)
>=4		1.23(0.87,1.75)		1.07(0.74,1.53)
Marital status				
Single		1		1
Married		1.78(1.09,2.89)		1.56(0.96,2.53)
Visited by the field worker				
No		1		1
Yes		1.29(0.95,1.75)		1.22(0.90,1.65)
Region				
Banjul			1	1
Kanifing			0.93(0.52,1.68)	0.99(0.54,1.84,)
Brikama			1.11(0.64,1.91)	1.14(0.64,2.02)
Mansakonko			0.96(0.46,2.01)	0.87(0.40,1.87)
Kerewan			2.96(1.35,6.49)	2.99(1.32,6.78) *
Kuntaur			1.81(0.84,3.89)	1.74(0.78,3.88)
Janjanbureh			1.18(0.57,2.44)	1.04(0.48,2.23)
Basse			2.02(0.97,4.18)	1.87(0.86,4.04)
Community-level women education				
Low			1	1
High			0.84(0.61,1.16)	0.84(0.60,1.18)
Community postnatal check				
Low			1	1
High			1.44(1.06,1.95)	1.48(1.08,2.02) *
Community-level women’s wealth status				
Low			1	1
High			0.78(0.50,1.20)	1.08(0.65,1.79)
The community heard family planning from HCPs				
Low			1	1
High			1.23(0.91,1.65)	1.23(0.91,1.66)
Place of residence				
Urban			1	1
Rural			0.98(0.58,1.66)	1.02(0.59,1.77)
Random effect/ measures of variation and model fitness/				
Community-level variance	0.45(0.25,0.81)	0.43(0.23,0.82)	0.24(0.11,0.53)	0.23(0.09,0.54)
ICC (%)	12.05	11.67	6.67	6.52
MOR (95% CI)	1.90(1.54,2.26)	1.88(1.50,2.25)	1.59(1.29,1.89)	1.58(1.27,1.89)
PCV (%)	Reference	4.45	46.67	48.89
LR	-974.07	-933.42	-948.97	-909.17
DIC (2LLR)	1,948.14	1,866.84	1,897.94	1,818.34
AIC	1952.14	1910.84	1925.95	1886.34

### Random effect and model comparison

The random effect outcome of diarrheal treatment-seeking behavior was shown in Table 3. The difference between clusters was responsible for 12.05% of the overall variation in the treatment-seeking behavior of diarrhea, according to the ICC value in the null model. Additionally, the null model’s MOR of 1.90 illustrates that there was a significant difference between clusters. This means when randomly selecting an individual from two different clusters (one cluster with a higher treatment-seeking behavior and the other cluster with lower treatment-seeking behavior), individuals in the cluster with a higher treatment-seeking behavior of diarrhea had 1.90 times higher odds of having treatment-seeking behavior as compared with their counterparts. According to PCV, the final model (model 3), which integrates both individual and community level characteristics, predicted 48.8% of the difference in low treatment-seeking behavior. All three parameters revealed that the effect of clustering was significant. Regarding model fitness, the best fit model was the final model (which had the lowest deviance) (Table 3).

## Discussion

This investigation aimed to explore the status of treatment-seeking behaviors and determinants among mothers of under-five children, and it declared that the overall treatment-seeking behavior is only 62.24% (95% CI: 59.67,64.74) of mothers have shown treatment-seeking behavior in their sick under-five children to brought them to the health facility. This prevalence is higher than a study conducted in Ethiopia 43% [35]. On the other hand, this result is lower than several earlier pieces of research in Gambia 81.5% [36], Bangladesh 75.16% [37], and Zambia 75.2% [38], that looked at how mothers in various locations sought out medical attention for common pediatric diarrhea. For several reasons, including participants’ treatment-seeking behavior for childhood disorders and their awareness of the ailment, this study’s real findings diverge from the one described above. One could come from a country’s profile of its childhood communicable disease and maternity health-related strategies. On the other hand, it can entail minimizing confounding variables, sample size, components included in the analysis with the type of analytic models, sociodemographic data about the respondents, time frame, latrine coverage, and use that may favorably or unfavorably affect the result variable.

In the regression analysis concerning the sex of children, the treatment-seeking behavior of mothers has shown reductions for female children. This finding is similar to studies conducted in Ethiopia, Uganda, and India [39–41]. Females were less likely to seek treatment early, according to a study on care seeking and access to health care for childhood illnesses published by Global DHS, and from various developing countries, thus females had a disadvantage in this [42, 43]. The concept of this discovery could be explained by the fact that gender inequality, which systematically harms and marginalizes women in the community, maybe the cause of the disparity in how mothers care for their male and female children. Because of their sociological and cultural viewpoints, male children have long drawn the attention of their parents and communities in Africa. Having male children has even occasionally been viewed as a [43–45]. This research aids poor nations in establishing intervention methods, such as health education and gender bias in seeking medical care. In contrast, this study contrasts with research done in Niger, Sierra Leone, and the Gambia in that gender wasn’t linked to treatment-seeking [36, 46, 47].

The other potent variable was household wealth status which has shown statistically significant in diarrheal treatment seeking attention. Mothers with middle and rich household incomes have a greater need for medical attention, while children are feeling sick. This figure is in agreement with other similar kinds of literature conducted in [48, 49], and global DHS trend [42]. This significance could be because, mothers with a better source of income have access to a better lifestyle, hygiene, feeding habit, and social media. All these in collective will have a significant effect on the prevention of infections like treatment-seeking behavior. In similar speaking, wealthy mothers are less concerned about the direct and indirect cost of medical care, so they are more likely to go to a health facility and treat their children timely and frequently than poor ones. Therefore, to improve child health, policymakers, planners, implementers, and other interested parties in the field of maternal and child health care should develop intervention strategies that promote the use of health insurance packages in the delivery of health services, accessibility to health care coverage, and free services for children under five in public health facilities.

Compared to mothers whose children were considered to be the average size at birth, mothers whose children were perceived to be smaller, and larger than usual at birth were more likely to seek medical attention for childhood illnesses. A study from 31 sub-Saharan African countries shows that smaller weighted babies have received more attention while they are getting [50]. This finding contradicted a study done in [51], however, we could not find more similar studies related to this discovery. This finding could be justified by mothers who gave birth to children of smaller weight may be able to take their children to the health facility when they are getting sick, as they may not think their children are healthy and safe due to their weight. In other words of expression, a mother who has given birth to a child with low birth weight is more likely to go to a health facility because of the health professional’s advice or because diarrhea can be very serious with them. In contrast, mothers whose children were perceived to be larger than usual at birth might have various perspectives regarding baby weight and physical growth. Babies who are considered larger in their weight at birth also are more likely to be physically overweight or appear older than their age, which their mothers may associate with illness or unhealthiness, and may be more likely to go to the health facility. On the other hand, mothers may relate it to another unknown situation depending on their knowledge, attitudes, and cultural practices. For this, they use traditional medicine and modern medical options from healthcare providers.

Radio media exposure has also shown a positive effect on mothers’ medical care-seeking behaviors. Mothers who have been exposed to radio media exposure has more likelihood of attention to medical care. This outcome has been agreed with other studies done in various places such as [52], [48], and [53]. As mothers follow media, they become increasingly monitor their children’s health, aware that their children’s health needs to be taken to a health facility as soon as possible. They also feed children a healthy diet, and symptoms of common childhood diseases, and more likely to know exactly where to get the treatment. Therefore, developing countries could bring about significant changes in their population by utilizing various forms of mass media, as media exposure is one of the best tools for enhancing the positive treatment-seeking behavior of people in resource-limited contexts.

Parents of children who have developed cough and fever have shown a higher tendency of medical care-seeking attention take to their children to health facilities than their counterparts. Evidence indicates that the number of individuals seeking treatment in healthcare facilities may have increased as illness severity [54]. Similar to other studies, our findings also show that detecting the severity of disease by warning symptoms of fever, cough, and/or vomiting increases the likelihood of seeking treatment at the health care [55]. Higher educational attainment and higher household wealth are frequently linked to the ability to spot danger indications and the willingness to seek treatment. According to several studies, perceived sickness severity was not a reliable indicator of mothers’ or caregivers’ desire to seek care, which runs counter to our [56]. A growing body of factual data, however, reveals those children in low- and middle-income nations were more likely to obtain care if their moms or caregivers thought their sickness was [49, 55].

The study also reveals a significant association between heard information about oral rehydration of mothers and treatment-seeking behavior of childhood diarrhea compared to their counterparts. The likelihood of using oral rehydration among informed mothers is high. This evidence is similar to other plenty of studies done in [57], [58, 59], [37], and sub-Saharan [60]. These mothers can use home remedies when their children are sick with diarrhea because they already know about the disease and know to treat it at home by giving various homemade medicines and consulting nearby professionals. Realizing this, these mothers may go to the health facilities immediately to treat their children, as they may be better educated, live better, have easy access to health facilities at their nearby, have more media exposure, and be the residents of the urban area. A significant improvement in the treatment-seeking behavior for childhood diseases will result from the dissemination of knowledge about oral rehydration and other homemade treatments for diarrhea and other childhood diseases through various mechanisms of coordination with healthcare providers, community leaders, traditional health educators, and other interested groups.

Mothers who have based their residence from region Kerewan have more positive attention to take their children to health facility while children have experienced than mother who is from Banjul region. Since the hidden potential variables of all regions were not thoroughly analyzed, the authors are unable to explain this discovery and believe that it should be investigated in future studies, however, the authors as a researcher, this may be explained by regional sociocultural differences, disparities in healthcare accessibility, disparities in healthcare quality, the commitment of various stakeholders and the way of implementation of the maternal and child health care services.

The other vital community-level determinant was postnatal checkup after delivery. Mothers who have a postnatal checkup after they had delivered a baby have shown much higher medical attention to their children by the time children developed diarrhea than their [58, 61]. To improve both the health of the mothers and the health of their babies, it is essential to encourage women to use postnatal care services provided at medical facilities and to seek medical guidance. The results of this study revealed that children whose mothers had not completed a baby postnatal checkup at a health facility were less likely than those whose mothers had to seek medical attention for diarrhea.

This has generated several advantages and disadvantages. Since it was based on nationally representative data, its representativeness is its main strength. Additionally, it was founded on a suitable statistical technique (multilevel analysis) to accommodate the hierarchical structure of the data. Because it is the first of its kind, the study findings could serve as a benchmark for future researchers and assist policymakers, planners, implementors, and other concerned stakeholders about the mother and child health service continuum in making an informed decision. As a drawback, there may be a chance of remembering bias due to the retrospective nature of the measurement of the date of the treatment-seeking behavior. In addition, causation between the dependent and independent variables cannot be proved because the data are cross-sectional. Furthermore, because the analysis is based solely on the data set’s information, certain variables and confounding factors like unfavorable outcome complications, participants’ complaints made while seeking treatment at health facilities, and the accessibility, viability, and acceptability of high-quality medical care are not taken into account.

## Conclusions

Gambia’s most pressing public health concerns. In this study, both individual and community-level factors were associated with treatment-seeking behavior of diarrhea. Among individual-level factors; having a female child, average weight baby at birth, the child had a cough and fever, mother hearing about oral rehydration, having radio media exposure, being from the middle and richer household wealth index, were associated with diarrheal disease treatment seeking behavior. Of community-level factors, region (being in the Kerewan region), and being from a high community level of postnatal checkup was associated with higher treatment-seeking behavior of diarrhea. Therefore, it is better if health professionals, possible stakeholders, and policymakers give special attention to treatment seeking behavior of mothers, institutional delivery, appropriate distributions of maternal health services, and coordination with regional states in different regions, especially in areas that are far apart from the health facilities to minimize delayed or bad treatment seeking behavior. Strengthening mothers’ healthcare-seeking behavior and skills on home remedies, and childhood illnesses, advocating mass media exposure, assisting financially disadvantaged mothers, postnatal checkups after delivery for both mothers and babies, and designing timely policies and interventions are highly advisable in the country.

## Data Availability

All data concerning this study are accommodated and presented in this document. The detailed data set can be freely accessible from the www.dhsprogram.comwebsite.
